# Case Report: Should IGF-1R targeted therapy be revisited in Ewing sarcoma? a report of long-term complete response and review of the literature

**DOI:** 10.3389/fonc.2025.1667628

**Published:** 2025-11-19

**Authors:** Georgios M. Stergiopoulos, Brittany L. Siontis, Safia K. Ahmed, Judith Jebastin Thangaiah, Matthew T. Houdek, Thanh P. Ho, Scott H. Okuno, Steven I. Robinson

**Affiliations:** 1 Department of Molecular Medicine, Mayo Clinic, Rochester, MN, United States; 2 Department of Oncology, Mayo Clinic, Rochester, MN, United States; 3 Department of Radiation Oncology, Mayo Clinic, Phoenix, AZ, United States; 4 Department of Laboratory Medicine and Pathology, Mayo Clinic, Rochester, MN, United States; 5 Department of Orthopedic Surgery, Mayo Clinic, Rochester, MN, United States

**Keywords:** case report, Ewing, IGF-1R, figitumumab, pegvisomant

## Abstract

**Introduction:**

Ewing sarcoma (ES) is a malignancy that mostly affects adolescents and young adults, with relapse or refractory cases posing major therapeutic challenges. Its unique transcriptional profile offers multiple targetable pathways, including the insulin-like growth factor-1 (IGF-1) receptor (IGF-1R) pathway.

**Case Report:**

We present the case of a 42-year-old female with recurrent ES with pulmonary metastases who, after progressing on anti-IGF-1R monotherapy with figitumumab (CP-751,871, NCT00560235), achieved complete remission in a phase I clinical trial (NCT00976508) that combined figitumumab IGF-1R-inhibition with growth hormone receptor antagonist pegvisomant. The patient has remained in long-term remission (>10 years) since the discontinuation of both agents and has not received any additional therapeutic interventions.

**Literature Review:**

We reviewed PubMed and the ClinicalTrials.gov database to identify clinical trials employing IGF-1R-targeted therapies in patients with ES and identified 24 relevant studies treating 723 patients with anti-IGF-1R therapy.

**Conclusion:**

This case represents the first report to our knowledge of patient outcomes following IGF-1R and growth hormone inhibition combination. The impressive response observed highlights the clinical synergy of this combination which warrants further clinical exploration as well as the potential of IGF-1R inhibition for ES. Additionally, this case suggests that targeted therapy discontinuation might be an option for select patients with long-term complete remission.

## Introduction

A better understanding of the physiology of cancer growth and progression has led to the identification of molecular targets and the development of effective targeted therapies, revolutionizing the treatment of solid and hematological malignancies (1). This progress extends to sarcomas, as an increasing proportion of these tumors appear to be driven by specific signaling pathways that could serve as novel therapeutic targets (1–6).

Ewing sarcoma (ES), the second most common malignant bone tumor in adolescents and young adults, is characterized by a high recurrence rate, frequent development of multi-drug resistance, and therefore poor survival following relapse (7, 8). ES is caused by pathognomonic translocations juxtaposing the EWS RNA binding protein 1 (EWSR1) gene with one of E26 transformation-specific (ETS) genes, with EWSR1 being most commonly fused with friend leukemia integration 1 (FLI1) or ETS-related gene (ERG) (9, 10). These typical translocations can alter the transcription of multiple gene activating pathways critical for oncogenesis and metastasis (11).

The insulin-like growth factor-1 (IGF-1) receptor (IGF-1R) pathway is among the dysregulated pathways affected by the EWS-FLI1 fusion. Its persistent activation has been demonstrated in ES cell lines and clinical samples, suggesting a key role in disease pathogenesis (12–14). Consequently, multiple clinical attempts have been made to inhibit IGF-1R signaling (15–33) with collective analysis of data from phase I and II clinical trials targeting this pathway in ES, having demonstrated a response rate of 10-14% (34, 35). Figitumumab (previously known as CP-751,871), a fully humanized monoclonal antibody (mAb) targeting IGF-1R, has been investigated as monotherapy and in combination with other agents in several phase I-III clinical trials (28, 31, 36–39).

We present a case of a patient with recurrent, refractory ES who achieved a complete response following treatment with dual IGF-1R and growth hormone receptor inhibition and review published literation on IGF-1R inhibition in ES. Additionally, we briefly present the efficacy and toxicity data of the NCT00976508 trial, which has not been published in a prior manuscript. This case is significant for multiple reasons. First, our patient’s response highlights the therapeutic potential of IGF-1R inhibition in ES. Second, the fact that the patient had previously progressed on figitumumab monotherapy underscores the importance of addressing the compensatory upregulation of growth hormone caused by the loss of negative IGF-1R feedback in the pituitary and hypothalamus during anti-IGF-1R therapy (15, 40). Finally, the patient’s long-term sarcoma remission, sustained nearly a decade after discontinuation of both agents, contributes to the ongoing discussion regarding the optimal duration of targeted therapy in patients who achieve long-term complete response.

## Case description

**Figure 1:**
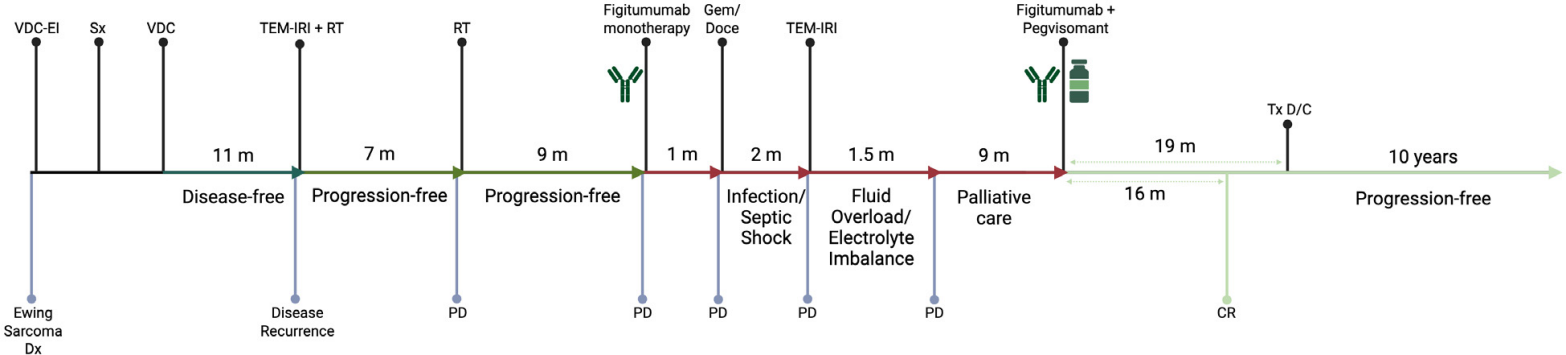
Timeline of different therapeutic interventions since the diagnosis of the primary Ewing Sarcoma tumor. CR, Complete Response; D/C, Discontinuation; Dx, Diagnosis; Gem/Doce, Gemcitabine and Docetaxel; m, month(s); PD, Progressive Disease; RT, Radiotherapy; Sx, Surgical Resection; TEM-IRI, Temozolomide and Irinotecan; Tx, Treatment; VDC, Vincristine, Doxorubicin, and Cyclophosphamide; VDC-EI, Vincristine, Doxorubicin, Cyclophosphamide, Etoposide/Ifosfamide.

We report the case of a 42-year-old female with recurrent ES who achieved complete remission following treatment with figitumumab and pegvisomant and has remained progression-free for >10 years after therapy cessation. A timeline of the different therapeutic interventions since the initial diagnosis is described below and presented schematically in **Figure 1**.

Our patient originally presented at the age of 35 with right shoulder pain. An x-ray revealed a proximal humerus mass which was biopsied and diagnosed as ES. She received neoadjuvant chemotherapy with vincristine, doxorubicin, and cyclophosphamide alternating with etoposide and ifosfamide followed by resection of the proximal right humerus. The patient subsequently received 11 cycles of adjuvant vincristine, doxorubicin and cyclophosphamide and remained disease-free for 11 months.

She developed metastatic disease, with left pulmonary mass, which was managed with 10 cycles of temozolomide, and irinotecan chemotherapy combined with radiotherapy, leading to the resolution of the mass. However, 7 months later the patient presented with Horner syndrome and left scapular pain, with imaging showing a left paravertebral/upper thoracic mass that was managed again with radiotherapy.

A follow-up computerized tomography (CT) scan 9 months later revealed bilateral pulmonary nodules biopsied positive for recurrent ES. She then participated in phase II arm of the phase I-II trial (NCT00560235/A4021020) with figitumumab monotherapy (4-week cycles, 30 mg/kg intravenously [IV] on days 1 and 2 first cycle subsequently, 30 mg/kg IV on day 1 for subsequent cycles). However, the patient developed disease progression during the first cycle and was switched to gemcitabine and docetaxel. After receiving a single cycle, she developed neutropenic fever complicated by septic shock. Following her recovery, she was found to have progressive disease and rechallenged with irinotecan and temozolomide. Due to rapid disease progression and decline in performance status, she was transitioned to palliative care.

**Figure 2:**
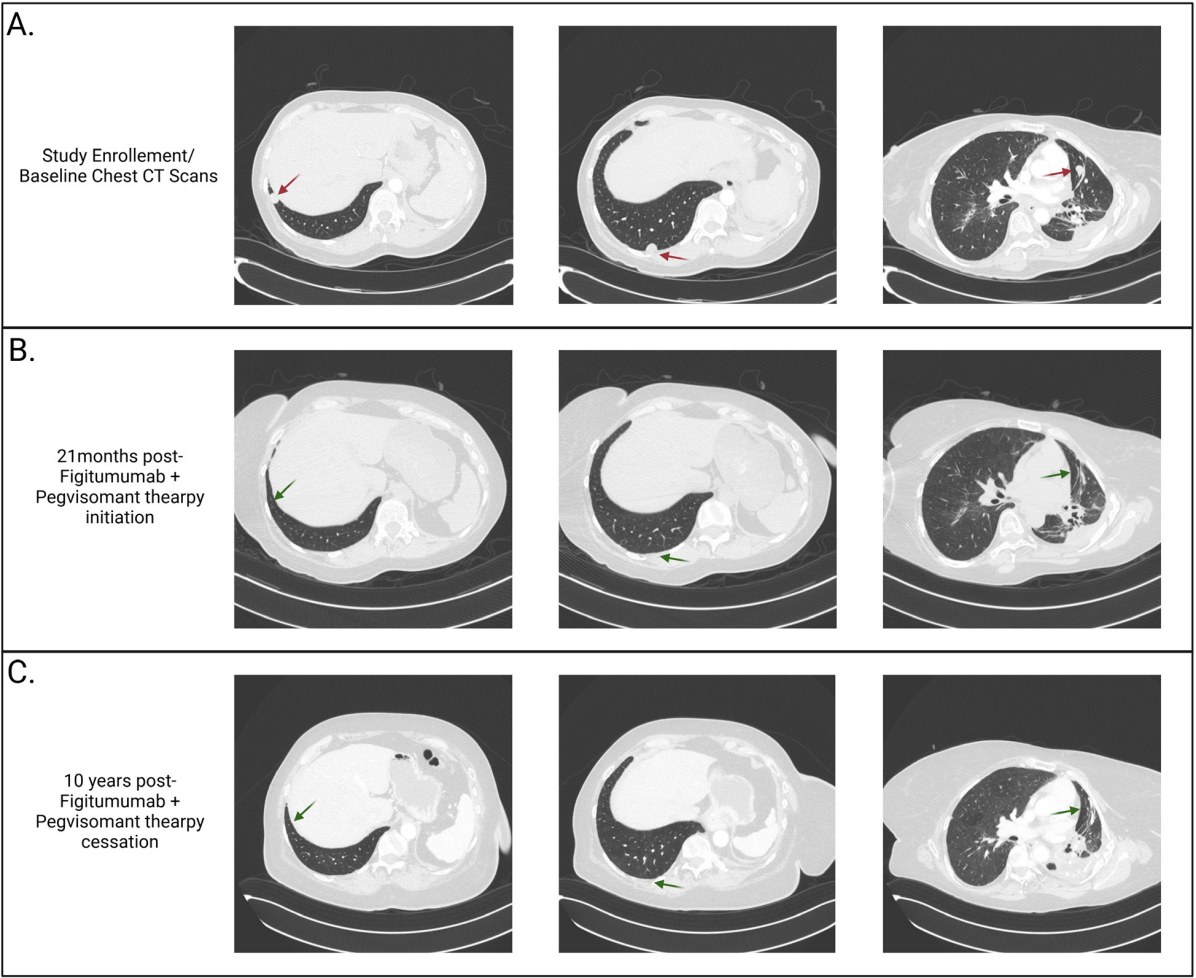
Computerized tomography (CT) scans. **(A)** Baseline CT scans of the lung demonstrating three intraparenchymal lung nodules (1.3 cm in diameter in the posterior and lateral right lower lobe, 1.4 cm in the subpleural right lower lobe, and 1.4 cm in the left mid-lung lobe) and post-radiation fibrosis (due to previous lines of therapy) prior to enrollment in the NCT00976508 clinical trial. **(B)** CT scans taken twenty-one months following clinical trial enrollment and two months following the completion of figitumumab and pegvisomant therapy, demonstrating complete resolution of the three lung nodules. **(C)** CT scans obtained ten years after discontinuing figitumumab and pegvisomant therapy, demonstrating sustained resolution of the lung nodules.

The patient had recurrent pulmonary infections but gradually improved over the ensuing 9 months. Remarkably, several of her lung nodules were either improved or stable, except for a growing right lung nodule (**Figure 2A**). Given her dramatic clinical improvement, she consented to participate in a phase I study (NCT00976508/A40201040) of combination therapy of figitumumab and pegvisomant. The treatment plan was structured in 3-week cycles, with the patient receiving 20 mg/kg of the anti-IGF-1R mAb figitumumab IV on day 1 and 20 mg/kg of the growth hormone receptor antagonist pegvisomant subcutaneously daily.

Following the closure of the clinical trial, she was put on a compassionate-use protocol (MC1212) to continue figitumumab and pegvisomant, and treatment resulted in sustained complete remission 16 months after therapy initiation. After 33 cycles (19 months) of therapy, which was tolerated well, she discontinued both agents due to the unavailability of figitumumab and entered the observation phase (**Figure 2B**). During treatment, the patient tolerated therapy well, with the main complaints being fatigue, two episodes of upper and lower respiratory tract infections, and a transient elevation in liver function tests. During the observation phase, she was monitored every three months for the first year, every four months for the second year, followed by six-monthly scans. She remains progression-free for >10 years after therapy cessation (**Figure 2C**).

## Literature review

The first preclinical data on IGF-1R inhibition in ES emerged two decades ago (13), however, multiple early-phase trials did not yield encouraging results, leading to the abandonment of single-agent IGF-1R mAbs as an experimental treatment option for ES.

We conducted a targeted literature review to identify clinical trials investigating anti-IGF-1R therapies in patients with ES. PubMed was searched using a combination of Medical Subject Headings (MeSH) and free-text terms for *sarcoma* and *Ewing sarcoma* together with variations of *IGF-1*, *IGF-1R*, and relevant therapeutic agents (ganitumab, linsitinib, cixutumumab, robatumumab, figitumumab, dalotuzumab). The search was restricted to clinical trial publications. This strategy yielded 34 articles, of which 19 reported the enrollment of at least one patient with ES and were therefore included in our review. In parallel, we searched the ClinicalTrials.gov database using an analogous set of terms to identify ongoing, or recently completed relevant trials that may not have resulted in peer-reviewed publications. This search identified an additional 6 trials. A detailed description of the search keys is provided in the **Appendix 1**, and the results are summarized in **Table 1**.

**Table 1:** Trials targeting IGF-1R for Ewing Sarcoma.

No	NCT	Phase	Disease	No of patients enrolled	Agent	Combinational therapy	Status	ES patient outcomes	Comments	Ref
1	NCT04199026	I (Early)	Multiple Sarcoma Subtypes (including ES)	20 (estimated)	Ganitumab (AMG 479, anti-IGF-1R mAb)	Microdosing with 9 additional agents (Doxorubicin, Ifosfamide, Vincristine, Irinotecan, Temozolomide, Pazopanib, Everolimus, Polyethylene glycol, and Temsirolimus)	Active, recruiting	N/A	Intratumoral drug delivery via implantable microdevice	N/A
2	NCT02306161	III	Newly diagnosed metastatic ES/PNET	298 (150 in the experimental arm)	Ganitumab (AMG 479, anti-IGF-1R mAb)	VDC-EI chemotherapy	Active, not recruiting (estimated completion: 2025)	The addition of Ganitumab was not associated with improved EFS survival, with 3-year EFS being 37.4% (95% CI: 29.3-45.5) for the standard arm and 39.1% (95% CI: 31.3-46.7) for the experimental arm Additional concerns for increased toxicity in the experimental arm (post-radiation pneumonitis, febrile neutropenia and transaminasemia)	RCT comparing chemotherapy ± anti-IGF-1R therapy	DuBois et al. (16)
3	NCT04129151	II	Relapsed/refractory ES	10	Ganitumab (AMG 479, anti-IGF-1R mAb)	Palbociclib	Terminated (2022)	ORR: 0% 3/10 patients had SD for >4 cycles and 2 had SD at completion of planned therapy/study closure	Terminated early due to discontinuation of ganitumab supply	Shulman et al. (17)
4	NCT02546544	II	Relapsed/refractory ES	16	Linsitinib (anti-IGF-1R kinase blocking)	–	Terminated (2016)	ORR: 0% 14/16 patients had PD	Did not reach estimated enrolment of 40 as 14/16 patients had PD	N/A
5	NCT01317420	I	Multiple Solid Tumors (including EFT)	64	Xentuzumab (BI836845, anti-IGF-1R mAb)	–	Completed (2016)	One patient with PNET had a PR (30.4 weeks)	Xentuzumab is well tolerated and showed preliminary anti-tumor activity	de Bono et al. (18)
6	NCT00642941	II	Relapsed/refractory EFT	115	R1507 (RG1507, anti-IGF-1R mAb)	–	Terminated (2014)	ORR: 10%, 10 PRs, 1 CR The median response duration was 29 weeks (range, 12–94 weeks)	R1507 is well tolerated and had a durable benefit in a subgroup of patients	Pappo et al. (19)
7	NCT01016015	II	Multiple Sarcomas (locally advanced, metastatic, or recurrent, including ES)	178 (27 ES/PNET)	Cixutumumab, (IMC-A12, anti-IGF-1R mAb)	Temsirolimus	Completed (2014)	ORR 15%, 4 PRs, no CR The mPFS was 7.5 weeks (95% CI: 5.6-17.7)	The combination showed clinical activity IGF-1R expression by IHC was not predictive of response to therapy	Schwartz et al. (20)
8	NCT01614795	II	Multiple Childhood/YA Sarcomas (Recurrent/refractory, including ES)	46 (12 ES)	Cixutumumab, (IMC-A12, anti-IGF-1R mAb)	Temsirolimus	Completed (2014)	ORR: 0%	N/A	(Wagner et al., 2015)
9	NCT00678769 (Expansion Cohort)	I	Refractory ES + DSRCT	20 (17 ES)	Cixutumumab, (IMC-A12, anti-IGF-1R mAb)	Temsirolimus	Completed (2013)	ORR: 12%, no PR, 2 CRs	1/6 ES patient who previously developed resistance to a different IGF-1R inhibitor antibody achieved a CR 7/20 patients (35%) achieved SD for >5 months or CR/PR	Naing et al. (22)
10	NCT00617890	II	ES + Osteosarcoma	219 (115 ES)	Robatumumab (SCH717454, MK-7454)	–	Completed (2013)	ORR: 7% 6 patients with metastatic ES who responded remained in remission for >4 years	–	Anderson et al. (23)
11	NCT00831844	II	Multiple Childhood Solid Tumors (recurrent/refractory, including ES)	116 (13 ES/PNET)	Cixutumumab, (IMC-A12, anti-IGF-1R mAb)	–	Completed (2013)	ORR: 15%	–	–
12	NCT01431547	I	Multiple Solid Tumors (including ES)	24 (6 ES)	Dalotuzumab (MK-0646, anti-IGF-1R mAb))	Ridaforolimus	Completed (2013)	ORR: 17%, 1 PR, no CR Time-to-response was 41 days and progression occurred at 126 days	–	Frappaz et al. (24)
13	NCT00563680	II	Metastatic EFT + DSRCT	38 (22 EFT)	Ganitumab (AMG 479, anti-IGF-1R mAb)	–	Completed (2012)	ORR: 5%, 1 PR, no CR	Ganitumab is well tolerated	Tap et al. (25)
14	–	I/II	Multiple Childhood Solid Tumors (including ES)	47 (35 ES/PNET)	Cixutumumab, (IMC-A12, anti-IGF-1R mAb)	–	Completed (2012)	ORR: 7%, 3 PRs, no CR	Cixutumumab is well tolerated in children as a single agent mPFS for highest dose cohort was 44 days (95% CI: 28-96)	Malempati et al. (26)
15	–	I	Multiple Solid Tumors (including ES)	27	AVE1642 (anti-IGF-1R mAb)	Docetaxel	Completed (2012)	1 PR	AVE1642 is well tolerated as a single agent and combined with docetaxel Promising activity in sarcoma and breast cancer	Soria et al. (27)
16	NCT00474760	I	Multiple Sarcoma Subtypes (including ES)	65 (16 ES)	Figitumumab (CP-751,871, anti-IGF-1R mAb)	–	Completed (2011)	ORR: 13%, 1 PR, 1 CR Figitumumab has antitumor activity in ES	Figitumumab is well tolerated	Olmos et al. (28)
17	NCT00400361	I	Multiple Solid Tumors (including ES)	37 (9 ES)	R1507 (RG1507, anti-IGF-1R mAb)	–	Completed (2011)	ORR: 22%, 2 PRs, no CR PRs were 11.5 and >26 months	–	Kurzrock et al. (29)
18	NCT00927966	I	Multiple Solid Tumors (including ES)	21 (1 ES)	Figitumumab (CP-751,871, anti-IGF-1R mAb)	Everolimus	Completed (2011)	ORR: 0%	Figitumumab + everolimus appear safe and well tolerated	Quek et al. (30)
19	NCT00976508	I	Multiple Solid Tumors (including ES)	23	Figitumumab (CP-751,871, anti-IGF-1R mAb)	Pegvisomant	Terminated (2011)	1 CR	Terminated early, not reaching planed enrolment of 42 patients	–
20	NCT00560235	I/II	ES, Osteosarcoma, and Other Sarcomas	138 (123 ES)	Figitumumab (CP-751,871, anti-IGF-1R mAb)	–	Completed-2010	ORR 12%, 15 PRs, no CR (in the phase II part) mPFS: 1.9 (95% CI: 1.8-2.8) mOS: 8.9 (95% CI: 7.2-11.1) (in the phase II part)	Figitumumab had modest activity as single agent in advanced ES Baseline free IGF-1 ≥ 0.65 ng/mL was associated with improved mOS (3.6 months vs 10.4 months, p=0.001)	Juergens et al. (31)
21	NCT00668148	II	Multiple Sarcomas (including EFT)	113 (18 EFT)	Cixutumumab, (IMC-A12, anti-IGF-1R mAb)	–	Completed (2010)	ORR: 6%, 1 PR, no CR (for EFT) PFR: 11% (for EFT) mPFS: 6.4 (95% CI: 5.1-12.1) months mOS 24.1 (95% CI: 12.6-37.6) months (for EFT)	Cixutumumab is well tolerated with limited toxicity	Schöffski et al. (2013)
22	NCT00701103	I	Multiple Solid Tumors (including ES)	80 (6 ES)	Dalotuzumab (MK-0646, anti-IGF-1R mAb)	–	Completed (2009)	ORR: 0% 1 patient with ES showed a mixed radiologic response	Dalotuzumab is well tolerated	Atzori et al. (33)
23	–	I	Multiple Solid Tumors (including ES)	53 (12 ES)	Ganitumab (AMG 479, anti-IGF-1R mAb)	–	Published (2009)	ORR: 17%, 1 PR, 1 CR	Ganitumab Is safe with absence of severe toxicities	Tolcher et al. (15)
24	NCT00609141	I	MultipleChildhood/YA Sarcomas (including ES)	34	Cixutumumab, (IMC-A12, anti-IGF-1R mAb)	–	Completed (2009)	–	–	–

CI, Confidence Interval; CR, Complete Response; DSRCT, Desmoplastic Small Round Cell Tumors; EFS, Event-Free Survival; EFS, Ewing Family of Tumors; ES, Ewing Sarcoma; IHC, Immunohistochemistry; mAb, Monoclonal Antibody; mOS, Median Overall Survival; mPFS, Median Progression-Free Survival; N/A, Not Available; ORR, Objective Response Rate; PFR, Progression-Free Rate; PD, Progressive Disease; PNET, Primary Neuroendocrine Tumor; PR, Partial Response; RCT, Randomized Controlled Trial; SD, Stable Disease; VDC-EI, Vincristine Doxorubicin, Cyclophosphamide, Etoposide/Ifosfamide; YA, Young Adults.

Among the 24 identified studies, 13 (54%) were phase I, 2 (8%) were phase I/II, 9 (38%) were phase II, and 1 (4%) was phase III. Most of these were safety and dose-finding studies enrolling patients with multiple advanced solid tumors and sarcomas, while only 4 (17%) studies focused exclusively on ES. The most common therapeutic modality targeting IGF-1R was mAbs, although one study (4%) investigated linsitinib, an orally administered small molecule that selectively targets IGF-1R and the insulin receptor. A total of 10 studies (42%) combined anti-IGF-1R mAbs with other agents, including mTOR inhibitors (n = 5, 21%), chemotherapy (n = 2, 8%), cyclin-dependent kinase (CDK) 4/6 inhibitors (n = 1, 4%), pegvisomant (n = 1, 4%), and one ongoing trial (4%) is exploring microdosing of 10 anti-tumor agents using an intratumoral device.

Across all trials, 723 patients with ES/Ewing Family of Tumors (EFT)/Primitive Neuroectodermal Tumor (PNET) were treated with anti-IGF-1R therapy. Of the 24 studies, 18 (75%) reported objective response rates (ORR) in patients with ES/EFT/PNET, covering approximately 573 patients. The reported ORR ranged from 0% to 17%, with a mean of 9%. Among the 52 responses described, 40 were partial responses, 5 were complete responses, and the remainder were unspecified.

Of note, clinical interest in the development and testing of these agents peaked during the previous decade, with 18 of the 24 studies (88%) conducted before 2016, and has since shown a marked decline in efforts to further evaluate their efficacy.

Regarding the only phase III clinical trial; it was run by the Children’s Oncology Group study and was terminated early in March 2019 after ganitumab (AMG479) failed to synergize with standard-of-care cytotoxic chemotherapy in newly diagnosed metastatic ES (16).

Despite these setbacks, the favorable safety profile and occasional complete and partial responses suggest that combination strategies warrant further exploration. One of the most promising approaches is the combination of IGF-1R with mTOR inhibition (16, 22, 34). This approach was designed following preclinical evidence of upregulation of IR-alpha, IRS-1, STAT3, MSTR1, and other proteins throughout the IGF-1R/PI3K/mTOR signaling cascade in xenografts that had adapted to IGF-1R-targeted therapy (41–44).

Notably, the first clinical trial combining the anti-IGF-1R mAb, cixutumumab, with the mTOR inhibitor, temsirolimus, demonstrated tumor regression of more than 20% in approximately 29% of the patients and a sevenfold increase in median response duration (>14 months) in patients with ES (22). Subsequent trials, however, failed to replicate an improvement in survival, with the results scrutinized due to concerns about the dosing of the mTOR inhibitor and the lack of comparative monotherapy arms in these single-arm studies (20). Nevertheless, a recent meta-analysis showed that combination of IGF-1R and mTOR inhibition improved progression-free survival (PFS) compared to IGF-1R inhibition alone in patients with ES (34).

By contrast, the available data on combining anti-IGF-1R therapy with CDK4/6 inhibitors remain limited and less promising. In one study exploring this combination, the ORR was 0%, with 3 of 10 patients (30%) achieving stable disease for more than 4 cycles and a 6-month PFS rate of 30% (17).

Finally, although biologically relevant, the combination of anti-IGF-1R mAbs with growth hormone inhibitors such as pegvisomant remains underexplored, with only one trial to our knowledge exploring this direction.

## Discussion

Compensatory growth hormone increase has been observed in patients treated with anti-IGF-1R mAbs since the introduction of these agents in the clinic (15). This is expected as anti-IGF-1R therapy disrupts the negative feedback loop at the hypothalamic–pituitary axis, leading to elevated circulating growth hormone which can upregulate IGF-1R expression and other downstream pathways that sustain tumor growth (45). Nonetheless, the combination of IGF-1R and growth hormone inhibitors, such as pegvisomant, has only been attempted in the clinical trial our patient was enrolled in (NCT00976508/A40201040).

This trial included two treatment arms: one with 10 mg/kg figitumumab plus 10 mg daily pegvisomant (arm A), and another with 10 mg/kg figitumumab plus 20 mg daily pegvisomant (arm B). Interestingly, none of the patients in the lower-dose pegvisomant group (n=0/17, 0%) achieved an objective response, whereas half of the patients in the higher-dose group (n=3/6, 50%) did, suggesting that a higher pegvisomant dose may be necessary to achieve therapeutic benefit.

Although our patient did not experience significant toxicity, serious adverse events were reported in 52.94% of patients in arm A (n=9/17) and 66.67% in arm B (n=4/6), underscoring the importance of carefully balancing efficacy with the risk of adverse effects. Reported toxicities included disease progression (n=5), gastrointestinal hemorrhage (n=1), death (n=1), pelvic infection (n=1), elevated blood uric acid (n=1), increased CRP (n=1), dehydration (n=1), back pain (n=1), flank pain (n=1), cauda equina syndrome (n=1), headache (n=1), and pneumonitis (n=1).

Despite the promising preliminary data, the study was terminated early on April 18, 2011. Termination was not due to safety concerns but related to timely recruitment in conjunction with the commercial discontinuation of figitumumab.

Our patient, who experienced disease progression on figitumumab monotherapy but achieved a sustained complete response with the combination of figitumumab and pegvisomant, demonstrates that growth hormone upregulation induced by IGF-1R-targeting mAbs can compromise the efficacy of this approach in the clinic. Additionally, clinical data from acromegaly therapy, which also targets this axis, suggest that pegvisomant, in combination with somatostatin analogs, is safe, effective, and can manage refractory acromegaly (46). This approach could also be explored in oncology, as it may offer a novel strategy to enhance treatment efficacy and overcome resistance to anti-IGF-1R therapies.

The above findings must be interpreted in the context of the challenges associated with identifying novel therapeutic targets and agents for ES. A meta-analysis of all phase I and II clinical trials enrolling patients with refractory or recurrent ES reported a median PFS of 1.9 months (range: 1.3–14.7) and an overall survival (OS) of 7.6 months (range: 5–30) (47). Only 18% of published trials were considered positive, with a median PFS of 4.5 months (range: 1.3–10) and OS of 16 months (range: 6.9–30) (47). Acknowledging the risk of publication bias, the actual outcomes may be even less favorable.

Furthermore, in IGF-1R–targeting phase I and II clinical trials, a subset of patients demonstrated significant clinical benefit, including our patient’s extraordinary long-term response, which raises the question of whether specific molecular or clinical features may have contributed. Although detailed molecular characterization of our patient’s tumor was not performed, certain predictive biomarkers have been identified in similar trials. Despite initial expectations, total IGF-1R expression in tumors has not been shown to predict response to IGF-1R therapy (20, 34, 48). By contrast, early data suggest that the absence of phosphorylated IGF-1R (pIGF-1R) (34) or exclusive nuclear staining of total IGF-1R (49) could serve as predictive biomarkers, potentially guiding future clinical trial enrollment. However, given the rarity of ES, implementing a precision medicine approach faces inherent challenges; limited patient populations restrict accrual in biomarker-enriched or stratified trials, underscoring the need for collaborative international consortia, basket trial designs, and adaptive methodologies.

Another critical topic for discussion in this case report is the optimal duration of targeted therapy for sarcomas. While targeted therapies are significantly less toxic than conventional chemotherapy (50, 51), adverse effects remain unavoidable. Common side effects include fatigue, rash, diarrhea, infections, hypertension, bleeding, thyroid dysfunction, proteinuria, and hepatotoxicity, which can range from mild to severe or even fatal (51–54). Additionally, there is often a disconnect between physicians’ perceptions and patients’ lived experiences. Many patients receiving small-molecule therapies deemed “well tolerated” by the medical community still report a significant decline in quality of life (55). Another factor to be acknowledged is the significant cost of these drugs, placing a heavy burden not only on patients themselves but also on social health resources (56, 57). This is only expected to increase in the coming years with the wider implementation of molecular treatments, coupled with the substantial inflation-adjusted price growth of targeted therapies over the past decades (57).

Although cases of long-term remission following discontinuation of targeted therapies have been reported, treatment cessation is rarely attempted due to concerns about disease recurrence. As a result, this field remains largely unexplored. Limited data on targeted therapy discontinuation come from patients with gastrointestinal stromal tumors (GISTs), where tyrosine kinase inhibitors (TKIs) such as imatinib form the cornerstone of treatment (58). Current guidelines recommend indefinite continuation of TKIs in the absence of disease progression (50). This approach is supported by clinical trial data showing that imatinib discontinuation, followed by re-initiation upon disease progression, is associated with decreased time-to-resistance and worse OS (59, 60). However, a significant proportion of patients in the discontinuation arm remained disease-free three years post-treatment cessation, suggesting that some individuals may be at lower risk for recurrence (59). A recent study proposed that minimal tumor burden may be a positive predictive factor for long-term remission following targeted therapy discontinuation—an observation that aligns with the case presented in this manuscript (61).

## Conclusions

Although clinical testing of IGF-1R-targeted therapies in ES has faced challenges, including the failure of single-agent approaches and the discontinuation of key studies, its potential remains significant, especially when combined with other agents. The combination of IGF-1R inhibitors with mTOR inhibitors or growth hormone receptor inhibitors, such as pegvisomant, represents an exciting avenue for further exploration, as demonstrated by the outstanding clinical response in our patient. Additionally, while the optimal duration of targeted therapy remains uncertain, the possibility of therapy discontinuation in select patients could be considered, as the long-term remission can sometimes be sustained. Refining patient selection through predictive biomarkers and exploring combination therapies could help maximize the efficacy of IGF-1R-targeted treatment and provide potential therapeutic options for patients with refractory or recurrent ES as well as other tumors. Notably, among the antibodies tested in ES, only ganitumab remains in active clinical development. Its pharmacological profile, which is comparable to figitumumab, suggests that it could be combined with growth hormone receptor antagonists to replicate or extend prior observations. While next-generation IGF-1R inhibitors would be valuable, prioritizing the evaluation of combinations using currently available, clinically approved, and safe agents may accelerate the generation of actionable data.

## Data Availability

The original contributions presented in the study are included in the article/**Supplementary Material**. Further inquiries can be directed to the corresponding author.
