# Supplementary material for: Case Report: Should IGF-1R targeted therapy be revisited in Ewing sarcoma? a report of long-term complete response and review of the literature
Source: Front Oncol. 2025 Nov 19;15:1667628. doi: 10.3389/fonc.2025.1667628 (PMC12672350; doi:10.3389/fonc.2025.1667628)
Supplement: Supplementary file 1 [file DataSheet1.docx]

**Appendix 1**

PubMed Query (last accessed September 26, 2025):

( (Sarcoma[MeSH Terms] OR Sarcoma[Title/Abstract] OR "Ewing Sarcoma"[MeSH Terms] OR "Ewing Sarcoma"[Title/Abstract]) ) AND ( ("Insulin-Like Growth Factor I Receptor"[MeSH Terms] OR IGF-1R[Title/Abstract] OR IGF1R[Title/Abstract] OR IGF-IR[Title/Abstract] OR "Insulin-Like Growth Factor 1 Receptor"[Title/Abstract]) OR ("Insulin-Like Growth Factor I"[MeSH Terms] OR IGF-1[Title/Abstract] OR IGF1[Title/Abstract] OR IGF-I[Title/Abstract] OR "Insulin-Like Growth Factor 1"[Title/Abstract] OR "Somatomedin C"[Title/Abstract]) OR (anti-IGF-1R[Title/Abstract] OR anti-IGF1R[Title/Abstract] OR anti-IGF-IR[Title/Abstract] OR "IGF1R inhibitor"[Title/Abstract] OR "IGF-1R inhibitor"[Title/Abstract] OR "IGF1R blockade"[Title/Abstract] OR "IGF1R monoclonal antibody"[Title/Abstract]) OR (Ganitumab[Title/Abstract] OR Linsitinib[Title/Abstract] OR Cixutumumab[Title/Abstract] OR Robatumumab[Title/Abstract] OR Figitumumab[Title/Abstract] OR Dalotuzumab[Title/Abstract]) ) AND (Clinical Trial[ptyp])

ClinicalTrials.gov Query under “other terms” (last accessed September 26, 2025):

(Sarcoma OR "Ewing Sarcoma") AND (IGF-1R OR IGF1R OR IGF-IR OR "Insulin-Like Growth Factor 1 Receptor" OR IGF-1 OR IGF1 OR IGF-I OR "Insulin-Like Growth Factor 1" OR "Somatomedin C" OR anti-IGF-1R OR anti-IGF1R OR anti-IGF-IR OR "IGF1R inhibitor" OR "IGF-1R inhibitor" OR "IGF1R blockade" OR "IGF1R monoclonal antibody" OR Ganitumab OR Linsitinib OR Cixutumumab OR Robatumumab OR Figitumumab OR Dalotuzumab)
